# The Lords' Amendments to the New Bill

**Published:** 1918-02-16

**Authors:** 


### NATIONAL INSURANCE ACT DEVELOPMENTS.

### The Lords' Amendments to the New Bill.

Recent events have once more clearly shown the immense value of the House of Lords as a check upon ill-considered legislation. The National Health Insurance Bill had received the assent and approval of the House of Commons, and in the course of its passage through that House it had been submitted to careful scrutiny by a Standing Committee—yet, in spite of all the care and attention that had been bestowed upon it, it was left to Lord Knutsford, ably supported by Lord Sydenham, to point out and rectify the most important defect in the Government's proposals.

### The Marriage Benefit.

It had been proposed under the Bill as originally drafted, and as agreed by the House of Commons, to set up a new benefit under the title "Marriage Benefit." This benefit was to take the form of a payment of £2 in cash to insured women who were members of approved societies upon giving notice to their societies within four weeks of the date of marriage of their desire to receive such payment in consideration of the surrender of their interests under their national health insurance.

The Government had—on the recommendation of the Ryan Committee—incorporated a proposal in the Bill which would in effect have provided "Marriage Dowries." The Government agreed and the House of Commons agreed, but the House of Lords disagreed. It was clearly felt—though perhaps not expressed in so many words—by the Upper House that those most in need of care and provision during sickness and maternity would be the very ones who would elect to take the cash payment on marriage, and would fritter it away in a manner that would not be conducive to their health or to the best interests of the community.

### The Amended Clause.

The Bill has now become an Act, though at the time of writing we are still without the text as finally approved. But from the report of the debate in the House of Lords on the Report stage it is possible to gather the terms of the section which makes special provisions with respect to married women. Stated briefly, the effect of the section is that if, upon her marriage, an insured woman who is a member of an approved society ceases to be employed she thereupon loses her right to the normal insurance benefits to which she would otherwise have been entitled. But instead of the normal benefits she is to be entitled, without paying any further contributions, to receive (1) a special sickness benefit at the rate of 5s. a week for not more than six weeks; (2) a maternity benefit of £1 10s. in respect of her first confinement within two years after her marriage; and (3) medical and sanatorium benefits. The benefits here numbered (1) and (3) are for varying periods, exactly defined in the Act, which have been designed to coincide with the insurance years of account. Full information on these points is to be printed on the insurance cards. It is understood that the section does not come into operation until July 1 next.

### Simplification of Administration Preserved.

It would appear, however, that there is no longer to be any optional choice of benefits for married women who do not remain in employment. There is to be one standard and one only, and provision is also made to eliminate the various existing special voluntary contributions by compensating them by means of surrender values. Thus from the point of view of the approved societies simplification would seem to have advanced one step further than had been contemplated in the Bill as originally drafted. The Act also provides penalties in the form of loss of benefit if notice of marriage is not given to the approved society within eight weeks.

The delay, brief though it was, that was entailed by the opposition of the House of Lords to the Marriage Benefit was fruitful in another way, for it afforded time for the careful reading of the measure after it had left the Commons. This careful reading elicited quite a host of minor drafting amendments, which the Government proposed and experienced little difficulty in carrying. The correction of the measure in these matters of detail will, we venture to think, be no less important than many apparently larger questions when it comes to the practical administration of the new Act. Thus once again the prime necessity for an alert revising Chamber—such as the House of Lords has proved itself to be—has been demonstrated.

### The Hospitals Clause Again.

It is necessary to record in conclusion that Lord Balfour of Burleigh endeavoured to secure the amendment of the Act in relation to the payment of sickness benefits to inmates of institutions. He raised the issue primarily with regard to the payment in respect of insured persons who are treated in Poor-Law infirmaries. But the question of payment of benefit to voluntary hospitals in respect of insured persons who have no dependants and for whom the sickness benefit is accumulated until their discharge from hospital was also mentioned, but without result. The Government takes the view that sickness benefit payments are sacred to the personal use of the insured person. Lord Sandhurst is reported as saying "there is nothing to prevent an insured person, if he chooses, from paying a sum of money or being allowed to pay a sum of money to a voluntary institution." Thus the voluntary hospitals are no better off than they were, and although evidence has been produced repeatedly showing the advantages which accrue to approved societies by the treatment of their members when seriously ill in the voluntary hospitals, yet the societies are permitted to accept the services of the institutions without in any way contributing to their funds. When will the societies recognise in a practical way the benefits they receive from institutions established long before the societies themselves were dreamed of?

